# HIV-1 nef suppression by virally encoded microRNA

**DOI:** 10.1186/1742-4690-1-44

**Published:** 2004-12-15

**Authors:** Shinya Omoto, Masafumi Ito, Yutaka Tsutsumi, Yuko Ichikawa, Harumi Okuyama, Ebiamadon Andi Brisibe, Nitin K Saksena, Yoichi R Fujii

**Affiliations:** 1 Molecular Biology and Retroviral Genetics Group, Graduate School of Pharmaceutical Sciences, Nagoya City University, Nagoya 467-8603, Japan; 2 Division of Nutritional Sciences, Graduate School of Pharmaceutical Sciences, Nagoya City University, Nagoya 467-8603, Japan; 3 Department of Molecular Diagnostics, Fields of Pathology, Nagoya University Graduate School of Medicine, Nagoya 464-8550, Japan; 4 Department of Pathology, Fujita Health University School of Medicine, Toyoake, Aichi 470-1192, Japan; 5 Research and Scientific Developments Division, Molecular Bio/Sciences Limited, 124 MCC Road, Calabar, Cross River State, Nigeria; 6 Retroviral Genetics Division, Center for Virus Research, Westmead Millennium Institute, Westmead Hospital, Westmead NSW 2145, Sydney, Australia

## Abstract

**Background:**

MicroRNAs (miRNAs) are 21~25-nucleotides (nt) long and interact with mRNAs to trigger either translational repression or RNA cleavage through RNA interference (RNAi), depending on the degree of complementarity with the target mRNAs. Our recent study has shown that HIV-1 *nef *dsRNA from AIDS patients who are long-term non-progressors (LTNPs) inhibited the transcription of HIV-1.

**Results:**

Here, we show the possibility that *nef*-derived miRNAs are produced in HIV-1 persistently infected cells. Furthermore, *nef *short hairpin RNA (shRNA) that corresponded to a predicted *nef *miRNA (~25 nt, miR-N367) can block HIV-1 Nef expression *in vitro *and the suppression by shRNA/miR-N367 would be related with low viremia in an LTNP (15-2-2). In the 15-2-2 model mice, the weight loss, which may be rendered by *nef *was also inhibited by shRNA/miR-N367 corresponding to suppression of *nef *expression *in vivo*.

**Conclusions:**

These data suggest that *nef*/U3 miRNAs produced in HIV-1-infected cells may suppress both Nef function and HIV-1 virulence through the RNAi pathway.

## Background

The human immunodeficiency virus (HIV), which infect humans cause acquired immunodeficiency syndrome (AIDS), which has reached pandemic levels in some societies, especially those in Southern Africa and Southeast Asia [1]. Given the immensity of HIV pandemic, the development of a rather safe and cheap, effective therapeutics, has become the main focus [2]. Several strategies attempted to control the spread of AIDS have not shown major breakthrough and the vaccines have shown little promise as far as their efficacy is concerned. However, one approach used extensively in other diploid organisms, which now has tremendous potential to encourage antiviral defense against HIV appears to be double stranded RNA-dependent post-transcriptional gene silencing or RNA interference (RNAi).

RNAi is a defense mechanism against aberrant transcripts that may be produced during viral infection and mobilization of transposons [3,4]. The RNAi pathway has been implicated in silencing transposons in the *C. elegans *germline [5,6], silencing stellate repeats in the *Drosophila *germline, and the response against invading viruses in plants [7]. Post-transcriptional regulation by RNAi is mediated by small non-coding RNAs (~25-nucleotides; nt). Small interfering RNAs (siRNAs) are short RNA duplexes that direct the degradation of homologous transcripts [8]. In contrast, the single stranded microRNAs (miRNAs) bind to 3' untranslated regions of mRNA with complementarity of 50 to 85% to give translational repression without target degradation [9]. The mature miRNA (~25-nt) is produced by processing of ~70-nt precursor stem-loop hairpin RNAs (Pre-miRNA) by Dicer [10,11]. At the moment several human diseases, including spinal muscular atrophy (Paushkin *et al*., 2002), fragile X mental retardation [13,14] and chronic lymphocytic leukemia [15] have been identified as illnesses in which miRNAs or their machinery might be implicated. However, up until now there has been no clear-cut scientific proof that establishes the exact correlation between miRNAs and human infectious diseases such as AIDS.

One of the human immunodeficiency virus type 1 (HIV-1) coding accessory genes, *nef*, is located at the 3' end of the viral genome and partially overlaps the 3'-long terminal repeat (LTR). The *nef *gene is uniquely conserved in HIV-1, HIV-2 and simian immunodeficiency virus (SIV) and is not essential but important for viral replication *in vivo *[16]. The *nef *gene is expressed during HIV infection and often accounts for up to 80% of HIV-1 specific RNA transcripts during the early stages of viral replication [2]. Our own investigations have shown that defective variants of *nef *dsRNA containing the 3'-LTR regions, obtained from long-term non-progressor (LTNP) AIDS patients, actually inhibited the transcription of HIV-1 [17]. Furthermore, *cis*-expression of mutated F12-HIV-1 *nef *inhibits replication of highly productive NL43-HIV-1 strain, which is not related to down-regulation of CD4 [18,19]. It has been demonstrated that F12 *nef *gene cloned from the provirus of naturally occurring HUT-78 T cells infected with the supernatant of the peripheral blood mononuclear cells (PBMCs) of an HIV-seropositive non-producer patient, induces a block of viral replication [19]. Thus, it has been suggested that *nef *RNAs may be a *cis*-regulatory factor for HIV-1 replication [20].

In the current study, we have established the link between miRNAs and HIV infections by demonstrating that *nef*-derived miRNAs are produced in HIV-1-infected cells. The results presented here show that *nef *short hairpin RNAs (shRNAs) corresponding to the *nef *miRNAs efficiently block RNA stability or mRNA translation, perhaps an indication that HIV-1 regulates its own replication by using *nef *miRNAs.

## Results and Discussion

### Identification of a candidate of miRNAs in HIV-1-infected cells

**Figure 1:**
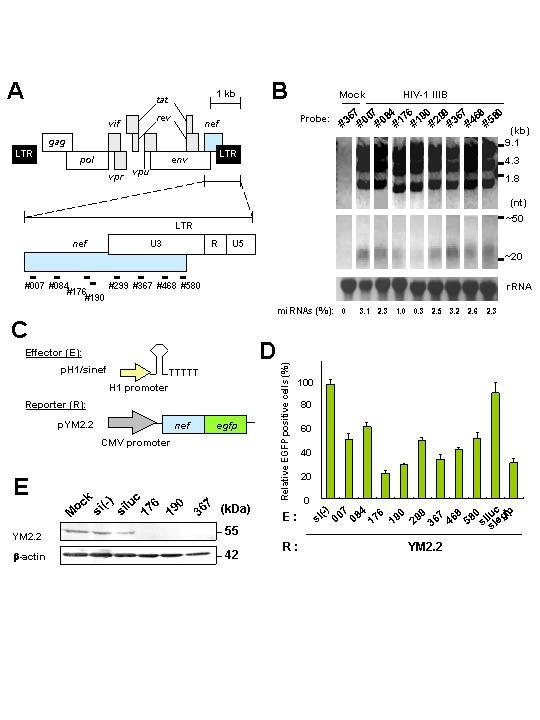
Detection of HIV-1 *nef *miRNAs and inhibition of Nef expression by *nef *RNAs. (**A**) Schematic representation of HIV-1 *nef *in its genome and synthetic DNA probes (#) used in this study. (**B**) Total RNAs were extracted from MT-4 T cells persistently infected with HIV-1 IIIB, separated on a 15% polyacrylamide-7 M urea gel, and subjected to northern blot analysis. The approximate sizes of the three classes of HIV-1 transcripts and small RNAs are indicated on the right. The loading control was rRNA stained with ethidium bromide. Relative expression (%) of *nef *small RNAs to the three classes of HIV-1 transcripts is at the bottom of figure. (**C**) Schematic representation of effector plasmids (**E**) H1 promoter-driven shRNA expression plasmids. Reporter (R) Nef-EGFP expression plasmid (pYM2.2) is also shown. **(D) **Inhibition by sinefs in pH1 plamids of Nef-EGFP expression. Either sinef, siluc or siegfp in each plasmid was transfected into Jurkat T cells in the presence of either pYM2.2 or control pEGFP-N1. At 36 h after transfection EGFP-positive cells were counted by flow cytometry. Data represent the relative activity of EGFP-positive cells where the percentage of positive cells in the sample transfected with pYM2.2 or pEGFP-N1 plus pcDNA3.1 or si(-) in pH1 plasmid was scored as 100%. Data are averages of three independent experiments + SD. Bars, SD. (**E**) Immunoblot analysis showing inhibition of YM2.2 expression by different *nef *shRNAs. Jurakat T cells were transfected with pYM2.2 and pH1/sinefs or siluc plasmid, cellular lysates were prepared 48 h after transfection, and immunoblotted with rabbit serum against Nef (upper panel) and anti-β-actin antibody (**lower panel**). The β-actin expression shows equal loading of all samples.

Very recently, the Epstein-Barr virus (EBV)-encoded miRNAs were identified. Thus, during the preliminary stages of this study, our curiosity was fixed on the need to find out if indeed there was any relationship between *nef *miRNAs and HIV-1-infected cells. To achieve this purpose, we extracted total RNA from HIV-1 IIIB strain persistently infected MT-4 T cells and northern blot analysis was performed using eight probes against the *nef *coding region, as shown in Figure 1A. Analyses using several anti-sense probes, small RNA molecules approximately ~25-nt in size were detected as well as HIV-1 major transcripts, 9.1, 4.3 and 1.8 kb bands (Fig. 1B). Similar results were obtained with total RNA from HIV-1 SF2 strain infected MT-4 T cells (data not shown). RNA samples treated with a mixture of the single stranded specific RNases A and T1 also generated ~25-nt RNAs that hybridized in northern blots with the sense probes against the same *nef *region. However HIV-1 major transcripts were not detected (data not shown), indicating that the structure of the small RNA molecules could be double-stranded RNAs (miRNAs). Some variability was observed when the quantity of the miRNAs was compared with the total of the major transcripts. A maximum of 3.2% of miRNAs was detected by using #367 probe when compared with total HIV-1 transcripts, and the minimum of 0.3% was detected by using probe# 90 (Fig. 1B).

To randomly clone the *nef *miRNA, ~25-nt RNAs were gel purified, cloned and sequenced. The sequences from the *nef *miRNA clones were 5'-acugaccuuuggauggugcuucaa-3' or similar ones, corresponded to the nucleotides approximately 420 to 443 conserved region of *nef *(miR-N367). The most notable feature of this analysis is that it has proven beyond reasonable doubts that *nef*-derived miRNAs are produced in HIV-1 infected cells.

### Inhibition of Nef by plasmids-encoding siRNA/miRNA

To examine inhibition of *nef *expression by the *nef *miRNA, we constructed eight shRNAs homologous to the native miRNA or probes used in Figure 1A[21]. Although it has been reported that three to four mutations in the sense strand derived from miRNA could have the potential to control unmutated 21-nt stem loop [22], we investigated whether the native shRNA-expressing plasmid can effectively reduce *nef *gene expression or not (Fig. 1C and 1D). We used *egfp *or *luc *gene (pH1/siegfp or luc) as a positive or negative control. All the shRNA-expressing plasmids including the controls were co-transfected into Jurkat T cells with pYM2.2 and cell fluorescence resulting from the expression of EGFP reporter gene was quantified by flow cytometry. The sinef176, 190, 367/miR-N367 and control siegfp all showed efficient reduction, but the sinef 007, 084, 299, 468 and 580 constructs gave only modest reductions, and no suppression was observed with si(-) and luc (Fig. 1D and Table 1). Immunoblot analysis using anti-Nef rabbit serum also confirmed the inhibition of Nef-EGFP expression by sinef 176, 190 and 367/miR-N367 (Fig. 1E).

**Table 1:** Relation between AIDS clinical courses and *nef *dsRNA or siRNA/miRNA in suppression of *nef *gene expression

Nef inhibitor	Nef expressed by:			Target region	Clinical courses
			
	Nef-EGFP	HIV-1 IIIB	PFV/nef		
dsRNA*					***Human***
SF2	++ ^†^	++	ND^‡^	Full-length (including miR-N367)	ND
1-3-3	++	-	ND	U3 deleted	Rapid progressor, Died within 3 years
4-2-1	ND	+	ND	U3 region (including miR-N367)	Rapid progressor, Died within 3 years
15-2-2	++	+++	ND	U3 region (including miR-N367)	Non progressor with low plasma viremia
16-1-1	ND	+	ND	U3 deleted	Non progressor with low plasma viremia
jw95-1	ND	+	ND	U3 region (including miR-N367)	Non progressor with undetectable viremia
***siRNA***					***15-2-2 model mouse***
dsnef	+	+	+	Full-length	ND
sinef007	+	ND	ND	Upstream of U3 region	ND
sinef084	+	ND	ND	Upstream of U3 region	ND
sinef176	+++	+++	++	Upstream of U3 region^§^	ND
sinef190	+++	+++	+++	Upstream of U3 region	ND
sinef299	+	ND	ND	U3 region (aa 83–135)	ND
sinef367/miR-N367	++	+++	++	U3 region	No weight loss
sinef468	+	ND	ND	U3 region	ND
sinef580	+	ND	ND	U3 region	ND

*dsRNA to LTNPs' *nef *has been described (Yamamoto *et al*., 2002).

^† ^-, negative; +, 0 to 50 % inhibition; ++, 50 to 75% inhibition; +++, 75 to 100 % inhibition.

^‡ ^ND, not done.

### Inhibition of Nef expression by STYLE vector-encoding nef siRNA/miR-N367

**Figure 2:**
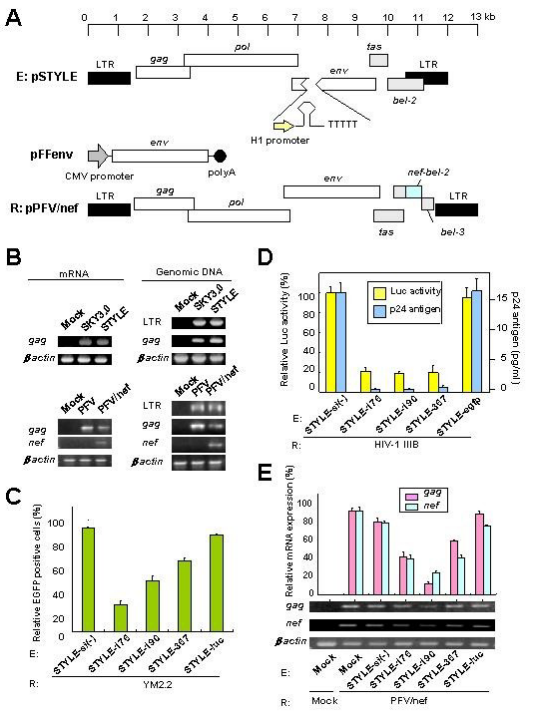
Inhibition of *nef *expression in human T cells by *nef *siRNAs. (**A**) Schematic representation of shRNA-expressing STYLE vector (**E**) and HIV-1 SF2 *nef *gene expressing pPFV/nef vector (R). The helper plasmid, pFFenv expresses FFV envelope protein under the control of CMV promoter. (**B**) Detection of expression of *nef *mRNA and integration of vectors. STYLE, SKY3.0, PFV/nef, PFV or mock was used to infect Jurkat T cells and the infected cells were cultured for 2 weeks. After 2 weeks, *gag *and *nef *mRNA expression was measured by RT-PCR. Genomic DNA of LTR, *gag *and *nef *of STYLE or PFV/nef were also detected by PCR. β-*actin *was used as a control. (**C**) Inhibition of Nef-EGFP expression by *nef *siRNA-expressing STYLE in Jurkat T cells. The pYM2.2 was transfected into each of the STYLE or mock-infected Jurkat T cells and EGFP-expressing cells were counted by flow cytometry at 48 h after transfection. Data represent the relative activity of EGFP-positive cells, where the percentage of positive cells in the sample transfected with pYM2.2 upon the STYLE-si(-) infected cells was scored as 100%. (**D**) Inhibition of HIV-1 transcription and replication by *nef *STYLE-367. HIV-1 IIIB persistently infected MT-4 T cells were transfected with the pLTR_SF2 _reporter and β-gal expressing control pCMVβ plasmids at 72 h after infection with STYLE. At 48 h post-transfection, Luc activity was measured and normalized as Luc values (Luc/β-gal). Absolute levels of Luc activity in the samples of pLTR_SF2 _plus SRYLE-si(-) were 16,311 + 1,253 or 783 + 87 light units for STYLE-367/miR-N367 transfectants. Data represent the relative Luc activities where the percentage of positive cells in the samples infected with the STYLE-si(-) was scored as 100%. After 48 h, p24 antigen was also measured in the cell culture supernatant of STYLE-infected Jurkat T cells. Data are averages of three independent experiments + SD. Bars, SD. (**E**) Inhibition of *nef *expression by *nef *siRNA in Jurkat T cells. Cells were infected with PFV/nef 48 h after infection with the STYLE and then subjected to semi-quantitative RT-PCR analysis. Data represent the relative expression of mRNA, where the percentage of positive cells in the sample of mock-infected cells (E: Mock) relative to the PFV/nef (R: PFV/nef) infected cells was scored as 100%. Data averages were derived from three independent experiments + SD. Bars, SD.

To assess the effects of *nef *miR-N367 *in vivo*, we constructed a prototype foamy virus (PFV)-based live vector. The PFV vector expressed HIV-1 SF2 *nef *gene as a reporter and the STYLE vector expressed shRNA as effectors. The full-length *nef *gene was inserted into the *bel-2 *portion of a PFV clone (22) in frame to obtain pPFV/nef (Fig. 2A). The pPFV/nef was transfected into BHK cells and treated with the histone deacetylase inhibitor, trichostatin A (TA) [23]. The viral supernatant, which contained approximately 5 × 10^6 ^infectious units (IFU), was collected at 72 h after transfection. For preparation of the STYLE vectors to deliver the shRNAs, the *env *gene portion of pSKY3.0 was replaced with the shRNA expression cassette under the control of the H1 promoter. The pSTYLE was produced (Fig. 2A), and transfected into the FFV envelope-expressing packaging cells, CRFK sugi clone # 6, in the presence of TA. The transfected CRFKsugi clone #6 had 99% FFV Env positive cells when analyzed by flow cytometry. The viral supernatant with a titer of ca. 1 × 10^5 ^IFU was collected at 72 h post-transfection.

The expression of viral mRNAs and integrated DNAs from either the PFV/nef or STYLE vectors was confirmed by infection of Jurkat T cells. The mRNAs and genomic DNA were extracted from the infected cells at 2 weeks post-infection. The PFV/nef-expressed *gag *and *nef *mRNAs and the STYLE-expressed *gag *mRNA were detected after amplification of these regions using reverse transcription (RT)-PCR. The integration of the DNAs into the genome of Jurkat T cells was also confirmed by PCR of the LTR, *gag *and/or *nef *regions (Fig. 2B). The control SKY3.0 and PFV-infected cells were both negative for *nef *mRNA and integrated DNA (Fig. 2B). The integrated DNA was also detected by southern blot analysis with genomic DNA of either PFV/nef or STYLE-infected cells (data not shown). Expression of shRNAs (~22-nt) was also confirmed in STYLE-infected cells by northern blot analysis (data not shown). Expression of Nef protein in PFV/nef-infected cells was also detected with specific rabbit anti-Nef serum in immunoblots (data not shown).

To evaluate whether the STYLE encoding siRNA could inhibit the expression of the *nef *gene in cultured human T cells, pYM2.2 was transfected into each of the STYLE-infected Jurkat T cells (m.o.i. = ca. 0.1). The most efficient sinef176, 190 and 367/miR-N367 vectors for reduction in *nef *expression (Fig. 1D and 1E) were selected for this experiment. The EGFP-positive cells were counted by flow cytometry at 48 h after transfection. Expression of Nef-EGFP fusion protein was reduced drastically following treatment with either the STYLE-176 (74 + 3.2) or 190 (51 + 4.2) and also reduced with 367/miR-N367 (32 + 2.3%). Reduction was insignificant with either the STYLE-si(-) (0 + 0.7) or STYLE-luc (7 + 0.9%) controls (Fig. 2C).

The *in vitro *inhibitory effects of STYLE encoding *nef *siRNA on HIV-1-infected cells were evaluated in Luc assays and using MT-4 T cells persistently infected with HIV-1 IIIB. Cultivation of the STYLE infected cells for 72 h followed by transfection with the pLTR_SF2 _and culture for another 48 h showed that STYLE-176, 190 and 367/miR-N367 all significantly (p < 0.005) suppressed Luc activity when compared to controls (Fig. 2D). HIV-1 p24 Gag was also significantly inhibited in the culture supernatant by infection with STYLE-176, 190 and 367/miR-N367 when compared to controls (p < 0.001) (Fig. 2D). These data suggested that shRNA/miR-N367 could inhibit HIV-1 transcription and replication in intact HIV-1-infected human T cells.

Jurkat T cells that had been transduced with *nef *shRNA for 48 h were infected with the PFV/nef. Semi-quantitative RT-PCR analysis revealed that while treatment with STYLE-190 dramatically reduced the expression of both *nef *and *gag *mRNAs of the PFV/nef, the expression of *nef *mRNA was also drastically suppressed by STYLE-176 and 367/miR-N367 (Fig. 2E). However the STYLE-si(-) and luc controls showed ~10% suppression of *nef *and ~20% suppression of *gag *mRNAs (Fig. 2E), which was probably a result of interference following super-infection. Nonetheless, both *nef *transcription and PFV/nef replication were substantially inhibited by STYLE-176, 190 and 367/miR-N367.

### Inhibition of Nef expression by siRNA/miR-N367 in mice

**Figure 3:**
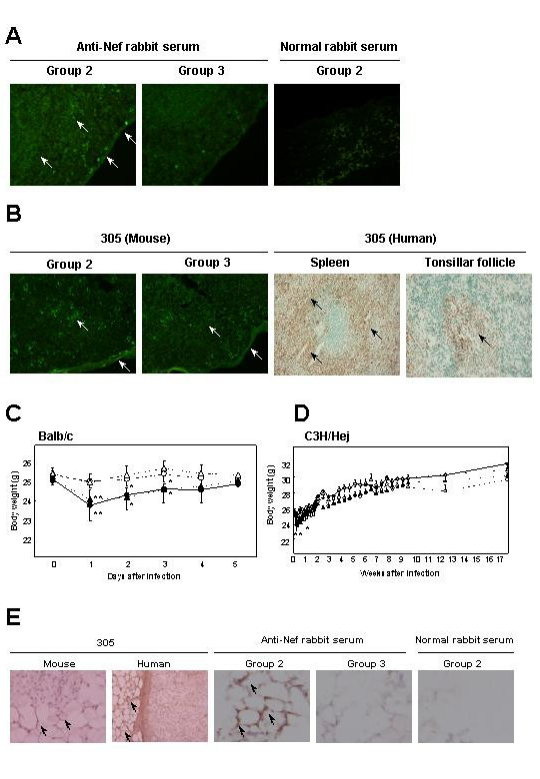
*In vivo *effects of miR-N367. (**A**) Distribution of Nef positive staining cells in the subcapsular area of groups 2 or 3 mouse spleens at 2 days after infection with PFV/nef. Anti-Nef rabbit serum or normal rabbit serum was used as a primary antibody. (**B**) Immunofluorescence for 305 mAb positive staining cells in the subcapsular area of groups 2 or 3 mouse spleens at 2 days after infection with PFV/nef and immunoperoxidase staining by 305 mAb in cells of interfollicular area of HIV-1 uninfected human spleen and tonsillar follicle. (**C**) Short term body weights of PFV/nef-infected Balb/c mice. The body weights of the PFV/nef-infected mice (group 1, n = 6, solid circle), the PFV/nef-infected followed by the STYLE-luc-infected mice (group 2, n = 6, solid triangle), the PFV/nef-infected followed by the STYLE-367-infected mice (group 3, n = 8, open triangle) and the STYLE-367-infected mice (group 4, n = 6, open circles) were measured from days 0 to 5. (**D**) Long term body weights of PFV/nef-infected C3H/Hej mice. Treatment of each group and numbers of mice were same as (**C**). Bars, SD. *; p < 0.05, **; p < 0.01 (relative to group 3). (**E**) Immunoperoxidase staining by 305 mAb and anti-Nef rabbit serum in cells of mouse or human adipose tissue. Arrows show positively stained areas. Magnification, X 20 (A and B); X 20 and X 200 (**E**).

Since different host gene products are required for siRNA-mediated RNAi and miRNA-mediated translational repression with *let-7 *and *lin-4 *in *C. elegans*, the two RNAs may not have the same functions *in vivo *[24]. To test this point, we investigated the efficacy of miR-N367 using STYLE-367 in mammalian tissues. The study mice were group 1 = PFV/nef-infected (n = 6); group 2 = PFV/nef and control STYLE-luc infected (n = 6); group 3 = PFV/nef and STYLE-367-infected (n = 8); and group 4 = STYLE-367-infected (n = 6). Identical study groups were used for both Balb/c and C3H/Hej mouse strains. Nef protein expressing lymphocytes were quantified by histochemical analysis using F3 Nef monoclonal antibody (mAb) or anti-Nef rabbit serum 2 days after PFV/nef infection. Nef protein was detected by immunofluoresence assay in the subcapsular area of the spleens of groups 1 or 2 Balb/c mice, but not groups 3 or 4 (Fig. 3A and Table 2). No positive cell staining was observed using normal rabbit serum as a primary antibody (Fig. 3A). To test the expression of *nef*, nested RT-PCR was also done on day 2 to evaluate the degree of *nef *mRNA expression in the spleen, liver, adipose tissues and hematopoietic cells in groups 1–4. The *nef *mRNA was significantly expressed in liver and hematopoietic cells of Balb/c mice in groups 1 and 2, but not in the group 3 animals that were STYLE-367 infected (Table 2). Tissues from group 4 did not show any *nef *bands after RT-PCR (data not shown).

**Table 2:** Histochemical detection and RT-PCR amplification of Nef from human and mouse tissues

Tissues	Histochemistry*			RT-PCR^†^		
	
	F3	Anti-Nef rabbit serum	305	*nef*	*gag*	*PPARγ*
*Human (HIV-1 uninfected)*						
Spleen	-^§^	-	+	-	-^‡^	ND
Tonsillar follicle	-	-	+	-	-	ND
Liver	-	-	+	-	-	ND
Adipose tissue (Salivary gland)	-	-	+	-	-	+
Bone marrow	-	-	+	-	-	ND
Bronchi	-	-	+	-	-	ND
Thyroid gland	-	-	+	-	-	ND
Heart muscle	-	-	+	-	-	ND
Prostate gland	-	-	+	-	-	ND
Testis	-	-	+	-	-	ND
Colon mucosa	-	-	+	-	-	ND
Lung	-	-	+	-	-	ND
Adrenal gland	-	-	+	-	-	ND
Brain (Cerebrum cortex)	-	-	+	-	-	ND
*Mouse Group 1 and 2*						
Spleen	+	+	+	+	+	ND
Liver	ND	ND	+	+	+	ND
Hematopoietic cells	ND	ND	+	+	+	ND
Adipose tissue (Intestine)	+	+	+	-	-	-
*Mouse Group 3*						
Spleen	-	-	+	±	±	ND
Liver	ND	ND	+	-	-	ND
Hematopoietic cells	ND	ND	+	-	-	ND
Adipose tissue (Intestine)	-	-	+	-	-	+

*Histological analysis was performed with human or each group of mouse tissues by using F3 anti-Nef mAb, anti-Nef rabbit serum or 305 mAb as a primary antibody. For secondary antibody, FITC or peroxidase-conjugated antibody was used.

^†^HIV-1 *nef*, PFV *gag *(^‡^HIV-1 *gag *for human tissues), and *PPARγ *mRNA expression were detected by RT-PCR with mRNA from human or each group of mouse tissues.

^§^+, positive; -, negative; ND, not done.

Because extracellular Nef is internalized into human and mouse lymphocytes and macrophages [25-27], we examined putative Nef receptor molecule (Ner) expression with 305 mAb [27] in both mouse and human tissues by histochemical analysis. In mice, 305 mAb positive lymphocytes were detected in the subcapsular area of the spleens by immunofluoresence assay (Fig. 3B) and liver and hematopoietic cells (Table 2) in groups 1, 2 and 3, indicating that detection of antigen by 305 mAb was not altered by Nef expression. In HIV-1 uninfected humans, the 305 mAb positive cells were detected by immunoperoxidase staining in spleen (red pulp), tonsillar follicle (germinal center), liver (Kupffer cells), salivary gland (germinal center and adipose cells), bronchi (smooth muscle cells), lung (stroma cells), thyroid gland (colloid), heart muscle (smooth muscle cells), prostate gland (smooth muscle cells), colon mucosa (intestinal absorptive and muscle cells), testis (basement membrane of tubuli seminiferi), adrenal gland (adipose cells), and brain (cerebrum cortex and cortical cells) (Fig. 3B and Table 2).

Since Nef suppressed PPARγ expression and reduced fatty acid levels *in vitro *[29-32], we monitored the expression of *PPARγ *mRNA and body weights of mice. Significant PPARγ mRNA expression in intestinal adipose tissue of group 3, but not group 1 and 2, was detected on day 2 (Table 2). All Balb/c mice in group 1 showed sedation and a drastic loss of weight from days 1 to 3 (day 1, p = 0.003; day 2, p = 0.021; day 3, p = 0.032 relative to mice in group 3) (Fig. 3C). Similar results were obtained in group 2 (Fig. 3C). However, group 3 mice infected with STYLE-367 did not appear to be sedated and had no drastic loss of weight (Fig. 3C). The group 4 animals, which were not infected with the PFV/nef but treated with STYLE-367, had no changes in either behavior or weight (Fig. 3C). In longitudinal examinations done during the post-infection period, the animals in groups 1 and 2 had recovered the lost weight (Fig. 3D). Similar results were obtained in group 2 from day 1 to 5 (day 1, p = 0.037; day 3, p = 0.044; day 5, p = 0.048 relative to mice in group 3) in the C3H/Hej mouse groups (Fig. 3D). To assess the above *in vivo *results, expression of *nef *mRNA was examined in adipose tissues (Table 2). As shown in Table 2, although mRNAs of *nef *and *gag *were not detected in mouse adipose tissues, 305 mAb and anti-Nef rabbit serum positive staining cells were detected in mouse group 1 and 2 adipose tissues (Fig. 3E and Table 2). Considering that the 305 mAb positive staining adipocytes appeared in mouse as well as human tissues (Fig. 3E and Table 2), these data suggest that the interaction between 305 and soluble Nef detected in adipose tissues may be responsible for the weight loss observed in mice.

In this study, whereas siRNA has been reported to inhibit hepatitis B virus replication *in vivo *(33–34), our results show that *nef*-derived miRNAs are produced in HIV-1 infected cells, and support the possibility that miRNA and siRNA may be functionally identical, at least in a retrotransposon such as HIV. Recent studies have revealed that miRNAs and siRNAs could block mRNA expression by similar mechanisms [9] and that siRNAs could function as miRNAs [35] and EBV-encoded miRNAs were found [36]. Our results reported here are consistent with these previous observations and are suggestive of the fact that *nef *miR-N367 could regulate *nef *expression even *in vivo*. In our unpublished data, HIV-1 LTR promoter activity was inhibited by miR-N367 (nt number 379 to 449 of SF2 *nef*, 71-nt) expression, of which activity was dependent on negative responsive element (NRE) of U3 region (our unpublished data). Although no mismatch shRNA against region #367 was active, the miR-N367 from HIV-1 genome may have some mismatches and effectively inhibit HIV-1 transcription. Further the effects of siRNAs of Tc*1*, in particular those to the terminal inverted repeats derived from read-through transcription of entire transposable elements, were presented for silencing transposase gene expression by RNAi machinery in germ lines of *C. elegans*, [37]. Taken together, it could be implied from these and our other results that miRNAs produced in HIV-1-infected cells can efficiently block not only Nef function but also HIV-1 replication through RNAi, which renders persistently low pathogenic infection latent as observed in an LTNP of 15-2-2 (see Table 1). It is equally important that although the weight loss reported here occurred only temporarily *in vivo*, however the inserted *nef *gene in the foamy retrotransposon may represent miRNAs which could inhibit *nef *mRNA expression by presumably an identical mechanism to that observed of siRNAs. Thus, RNAi might serve as a new sequence-specific therapeutic arsenal in AIDS prevention and possibly treatment.

Overall, our results indicate that nef shRNA transduced into T cell line inhibited HIV-1 transcription. Further, nef miRNAs could be produced from infected T cells and can block the trans-activity of Nef as well as HIV-1 replication on its own via the cis-action of nef. These functions of nef via RNAi pathways may allow persistently low pathogenic or latent infection as observed in HIV-infected non-progressors. Cumulatively, these data suggest that Nef may be involved in both viral replication and the disease progression, the findings, which may facilitate new strategies for HIV control *in vivo*.

## Materials and Methods

### Patient details

Patient selection is showed in Table 1. These SF2 (HIV-1 subtype B prototype) was included as a control *nef *sequence, because of the inclusion of viruses, which were also subtype B. The SF2 contained full-length *nef *reading frame as indicated in Table 1. Patients 1-3-3, 4-2-1 (Table 1) are rapid progressors infected with HIV-1. These patients were infected in 1984–1985 and died within 3 years of primary infection with >1 × 106 viral copies and CD4+ T cell count of 75 and 110/ml blood. Patients 15-2-2 and 16-1-1 (Table 1) are slow progressors, who were infected in 1984 and have survived HIV-1 infection with high and stable CD4+ T cell counts (690 and 760/ml blood) with low (<5000 copies) plasma viremia. All these patients acquired virus through homosexual sex. JW95-1 (Table 1) is a boy who was infected from his mother via breast feeding. The child was infected in 1983 and has survived disease free with high CD4+ T cell count (890/ml blood) with undetectable viremia. Human samples were obtained from a donor after informed consent.

### Cells and viruses

HeLa and BHK cells were grown in Dulbecco's modified Eagle Medium (DMEM) (GIBCO, Grand Island, NY) supplemented with 10% heat-inactivated fetal bovine serum (FBS) and antibiotics. CRFK cells were grown in Iscove's Modified Dulbecco's Medium (IMDM) (GIBCO) with 10% FBS and antibiotics. Jurkat T cells and MT-4 T cells persistently infected with HIV-1 IIIB strain were cultured in RPMI-1640 medium (GIBCO) supplemented with 10% FBS and antibiotics. The packaging cells (CRFKsugi) were made by transfecting CRFK cells with 10 μg of the pFFenv with Lipofectin Reagent (Invitrogen) and selecting transformants after culture for 14 days with 25 μg/ml of hygromycine B (Invitrogen). After 14 days, FFV Env protein expression was measured by flow cytometry and immunoblot analyses with FFV-infected cat B serum [34]. The pPFV/nef (10 μg) was transfected into BHK cells and pSTYLE/si (10 μg) was transfected into CRFKsugi cells with Lipofectin Reagent. The transfected cells were cultured for 72 hr, and the viral supernatant was collected and filtered through a 0.45 μm pore size Millex-GP filter (Millipore, Bedford, MA). Vector stocks were stored at -70°C prior to use. Viral titers were determined as described previously [21]. Cells were infected with PFV/nef and/or SKY/si at an m.o.i. of ca. 0.1 in the presence of 4 μg/ml of polybrene and infected cells were cultured at a density of 1 × 10^6 ^cells per ml for 3 days.

The details of plasmid constructs and the primer sequences used in cloning strategies are shown in supplementary file (see Additional file: 1).

### Flow cytometric analysis

Flow cytometry was performed with a FACS Calibur (Becton Dickson, San Jose, CA) as described previously [17].

### Luc assay and Immunoblotting

Firefly Luc assay was performed using the Luciferase Assay System (Promega) as described previously [17]. Immunoblotting was performed essentially as described previously by Otake et al. [28].

### P24 ELISA

The concentration of p24 supernatant was determined by an antigen capture assay (Beckman Coulter, Fullerton, CA) according to the manufacturer's instructions.

### Confocal laser microscopy analysis

Confocal laser microscopy analysis was performed as described previously [28].

### Northern blot analysis

Total RNAs were extracted from HIV-1 IIIB or SF2 persistently infected or uninfected MT-4 T cells using TRIzol reagent (Invitrogen). Approximately 40 μg of total RNA was treated with RNase A and T1 (Sigma, St. Louis, MO) as described previously [17], subjected to electrophoresis on a 15% polyacrylamide-7 M urea gel and electroblotted to HybondN+ (Pharmacia, Uppsala, Sweden) for 4 hr at 400 mA. RNAs were immobilized by UV crosslinking and baking for 1 hr at 80°C. Hybridization was done with an ECL direct Kit (Pharmacia). Synthetic DNA probes were labeled with horseradish peroxidase. The sequence for synthetic sense DNA probes for northern blot analysis are as follows: #007 (5'-gcgtcgacggcaagtggtcaaaacgta-3'); #084 (5'-gcgtcgacgccagcagcagatggggtg-3'); #176 (5'-gcgtcgacgtgcctggctagaagcaca-3'); #190 (5'-gcgtcgacgcacaagaggaggaga-3'); #299 (5'-gcgtcgacgactggaagggctaatttg-3'); #367 (5'-gctcgacggctacttccctgattggc-3'); #468 (5'-gcgtcgacggtagaagaggccaatgaa-3'); #580 (5'-gcgtcgacgcatttcatcacatggccc-3'). RNAs were cloned by 5'RACE System (Invitrogen, CA., USA) with a slight modification in that primers were used that were the same as the synthetic DNA probes as described above that are abbreviated as #primer. In brief, gel purified small RNAs were annealed with #primer and first strand cDNA was synthesized with SuperScript II RT (Invitrogen, CA., USA). Afrer RNase H and T1 treatment, a homopolymeric tail was added to the 3'-end of the cDNA using terminal deoxynucleotidyl transferase and dCTP. After ethanol precipitation, PCR amplification was done with abridged anchor primer and #primer. Then the PCR products were obtained using abridged universal amplification primer and #primer. The PCR fragments were digested with *Sal*I and cloned into *Sal*I site of pBluescript SK(-), followed by sequence analysis. The secondary structures of RNAs were predicted by GENETYX-MAC program (Software Development Co. Ltd, Tokyo, Japan).

### Semi-quantitative RT-PCR analysis

Semi-quantitative RT-PCR analysis was performed using the ThermoScript RT-PCR System (Invitrogen, CA., USA) according to the manufacturer's protocol with the following primers: III (5'-atcatgggccaaagagaattc-3') and IV (5'-aaatttcactcaatcgagcc-3') for FFV LTR, VI (5'-aggacctgaaaggcatg-3') and VII (5'-ttgttgagatcgtcccg-3') for FFV *gag*, VIII (5'-tgtggtggaatgccactag-3') and IX (5'-attgtcatggaattttgta-3') for PFV LTR, XI (5'-tcttacagaccagtaacaa-3') and XII (5'-gtcaatcattacatctgca-3') for PFV *gag*, XIII (5'-aactactagtacccttcagg-3') and XIV (5'-aaaactcttgctttatggcc-3') for HIV-1 *gag*, XV (5'-atgggtggcaagtcaaaacg-3') and XVI (5'-tcagcagtctttgtagtactccg-3') for HIV-1 *nef*, XVII (5'-gttatgggtgaaactctgggagat-3') and XVIII (5'-atgttcctgaacataatcgtc-3') for *PPARγ*, XIX (5'-gacaacggctccggcatgtgcaaag-3') and XX (5'-ttcacggttggccttagggttcag-3') for β-*actin*, respectively. The nested PCR followed RT reaction was performed as described previously [34]. PCR products were quantified with the NIH image program. Relative mRNA expression was calculated as percentage expression using the following formula: integrating number of *nef *or *gag *bands/integrating number of β-*actin *X 100.

### In vivo studies and tissue analyses

Balb/c and C3H/Hej mice were raised under specific pathogen-free (SPF) conditions. Mice were infected with 1 ml of 10^5 ^IFU of PFV/nef and STYLE-367 by intravenous (i.v.) injection. RT-PCR analyses were performed 2 days after infection. For histological analysis, cryostat sections were prepared from both human and mouse tissues. The fixed sections were rinsed with PBS and incubated with 5% BSA for at least 1 hr to inhibit nonspecific binding of antibodies. Sections were incubated overnight at 4°C with anti-Nef rabbit serum, F3 or 305 mAb, and incubated with peroxidase or FITC conjugated secondary antibodies. The washed sections were incubated in 0.03% 3,3'diaminobenzidine (Sigma) solution in 0.05 M Tris buffer with 0.01% H_2_O_2 _for development of peroxidase activity. After counterstaining with hematoxylin or methylgreen, the sections were dehydrated and mounted.

### Statistical methods

Data were analysed using a one-way ANOVA analysis with a post-hoc Fisher's test. P values of 0.05 or more were determined for that of cut off.

## List of abbreviations used

HIV-1 human immunodeficiency virus type 1

miRNA microRNA

nt, nucleotides

LTNP long-term non-progressors

shRNA short hairpin RNA

AIDS acquired immunodeficiency syndrome

RNAi RNA interference

siRNA small interfering RNA

LTR long terminal repeat

SIV simian immunodeficiency virus

PBMCs peripheral blood mononuclear cells;

EBV Epstein-Barr virus;

EGFP enhanced green fluorescence protein

PFV prototype foamy virus

TA trichostatin

A IFU infectious units

FFV feline foamy virus

RT reverse transcription

m.o.i., multiplicity of infectionm

Ab monoclonal antibody;

Ner Nef receptor molecule;

NRE negative responsive element.

## Competing interests

The authors declare that they have no competing interests.

## Authors' contributions

S.O. carried out northern analyses, immunoblot analyses, RNAi assays and was involved in the construction of plasmids. M.I. and Y.T. participated in *in vivo *studies and tissue analyses. Y.I. and H.O. participated in data validation and overall experimental design. E.A.B. and N.K.S. carried out the clinical, sequencing, and virological studies and the writing of the manuscript. Y.R.F. participated in the design of the study and coordinated it. All authors read and approved the final manuscript.

## Supplementary Material

Additional file 1 Click here for file
