# Supplementary material for: HIV-1 nef suppression by virally encoded microRNA
Source: Retrovirology. 2004 Dec 15;1:44. doi: 10.1186/1742-4690-1-44 (PMC544868; doi:10.1186/1742-4690-1-44)
Supplement: Additional file 1 [file 1742-4690-1-44-S1.doc]

**Supplementary material**

**Plasmid construction**

The human H1/promoter fragment was amplified by PCR from total DNA of Jurkat T cells and cloned into pGEM-T Easy TA cloning vector (Promega, Madison, WI) with primers C (5’-acgcgtcgacctgacgtcatcaacccgctccaagg-3’) and D (5’-aactgcaggctagcgtggtctcatacagaacttataag-3’) (pH1). Sense and antisense oligonucleotides bearing *Nhe*I and *Sac*II linker sequences for construction of pH1/sinef007, 084, 176, 190, 299, 367, 468, 580, luc or egfp were prepared as shown below. For 007, sense (5’-ggactagtgctagcggcaagtggtcaaaacgtagaaaatacgttttgaccacttgcctttttccgcggtctagagc-3’) and antisense (5’-gctctagaccgcggaaaaaggcaagtggtcaaaacgtattttctacgttttgaccacttgccgctagcactagtcc -3’); for 084, sense (5’-ggactagtgctagcgccagcagcagatggggtggaaaacaccccatctgctgctggctttttccgcggtctagagc-3’) and antisense (5’-gctctagaccgcggaaaaagccagcagcagatggggtgttttccaccccatctgctgctggcgctagcactagtcc-3’); for 176, sense (5’-ggactagtgctagcgtgcctggctagaagcacagaaaatgtgcttctagccaggcactttttccgcggtctagagc-3’) and antisense (5’-gctctagaccgcggaaaaagtgcctggctagaagcacattttctgtgcttctagccaggcacgctagcactagtcc -3’); for 190, sense (5’-ggactagtgctagcgcacaagaggaggaagagggaaaacctcttcctcctcttgtgctttttccgcggtctagagc-3’) and antisense (5’-gctctagaccgcggaaaaagcacaagaggaggaagaggttttccctcttcctcctcttgtgcgctagcactagtcc-3’) ; for 299, sense (5’-ggactagtgctagcgactggaagggctaatttggaaaacaaattagcccttccagtctttttccgcggtctagagc-3’) and antisense (5’-gctctagaccgcggaaaaagactggaagggctaatttgttttccaaattagcccttccagtcgctagcactagtcc-3’); for 367, sense (5’-ggactagtgctagcagcaccatccaaaggtcaggaaaactgacctttggatggtgcttttttccgcggtctagagc -3’) and antisense (5’-gctctagaccgcggaaaaaagcaccatccaaaggtcagttttcctgacctttggatggtgctgctagcactagtcc--3’); for 468, sense (5’-ggactagtgctagcggtagaagaggccaatgaagaaaattcattggcctcttctacctttttccgcggtctagagc-3’) and antisense (5’-gctctagaccgcggaaaaaggtagaagaggccaatgaattttcttcattggcctcttctaccgctagcactagtcc-3’); for 580, sense (5’-ggactagtgctagcgcatttcatcacatggcccgaaaagggccatgtgatgaaatgctttttccgcggtctagagc-3’) and antisense (5’-gctctagaccgcggaaaaagcatttcatcacatggcccttttcgggccatgtgatgaaatgcgctagcactagtcc-3’); for luc, sense (5’-actagctagccacttacgctgagtacttcgaaaagaagtactcagcgtaagtgtttttccgcggggat-3’) and antisense (5’-atccccgcggaaaaacacttacgctgagtacttcttttcgaagtactcagcgtaagtggctagctagt -3’) ; for egfp, sense (5’-ggactagtgctagcggctacgtccaggagcgcagaaaatgcgctcctggacgtagcctttttccgcggtctagagc-3’) and antisense (5’-gctctagaccgcggaaaaaggctacgtccaggagcgcattttctgcgctcctggacgtagccgctagcactagtcc-3’). Each oligonucleotide was annealed, digested with *Nhe*I and *Sac*II and cloned into pH1 digested with the same enzymes. For insertion of the human H1 promoter plus shRNAs fragment into pSKY3.0 (Hatama *et al*., 2001b), the fragment was PCR-amplified by primers E (5’-acgcgtcgactcatcctgactgacgtcatcaacccgctcc-3’) and F (5’-taatacgactcactataggg-3’). The amplified fragment was blunted with T4 DNA polymerase, digested with *Sal*I, and ligated into the pSKY3.0 after digestion with *Sal*I and *Hpa*I (pSTYLE).

For preparation of the FFV envelope protein expressing plasmid, the full-length *env* fragment was amplified by PCR with primers G (5’-ttagcggccgctatggaacaagaacatgtgatg-3’) and H (5’-gctctagattactggtccttcttccggg-3’) using pSKY3.0 as a template. The amplified fragment was then digested with *Not*I and *Xba*I after which the *env* fragment was ligated into the pcDNA3.1/Hyg vector digested with the same enzymes (pFFenv). To construct pPFV/nef, full-length SF2 *nef* was amplified by PCR with primers I (5’-acatgcatgcatgggtggcaagtggtcaaaacg-3’) and J (5’-acatgcatgctcagcagtctttgtagtactccg-3’) using SF2 plasmid as a template. The amplified fragment was digested with *Sph*I and inserted into the *Sph*I site of pHS007 as described previously (Fujii *et al*., 2004). Preparation of the HIV-1 SF2 LTR-luc reporter plasmid (pLTR_SF2_), Nef-fused EGFP expressing plasmid (pYM2.2) and expression vectors of PPARγ (pSVSPORT-PPARγ2) was described previously (Yamamoto *et al*., 2002; Otake *et al*., 2004).
